# *Lactobacillus*-Fermented *Centella asiatica* Extract Inhibits Airway Inflammation in Cigarette Smoke Extract/LPS-Induced Mice

**DOI:** 10.3390/plants14223416

**Published:** 2025-11-07

**Authors:** Yoon-Young Sung, Eunjung Son, Dong-Seon Kim, Seung-Hyung Kim, Won-Kyung Yang, Misun Kim

**Affiliations:** 1 KM Science Research Division, Korea Institute of Oriental Medicine, Daejeon 34054, Republic of Korea; ejson@kiom.re.kr (E.S.); dskim@kiom.re.kr (D.-S.K.); 2 DJU Industry-University Cooperation Foundation, Daejeon University, Daejeon 34520, Republic of Korea; sksh518@dju.kr; 3 Institute of Traditional Medicine and Bioscience, Daejeon University, Daejeon 34520, Republic of Korea; ywks1220@dju.kr; 4 Bio Technology R&D Center, WiLab Co., Ltd., Seoul 04789, Republic of Korea; kms@wilaboratory.com

**Keywords:** *Centella asiatica*, fermentation, COPD, cytokines, bronchoalveolar lavage fluid

## Abstract

*Centella asiatica*, a widely used medicinal herb in Oriental and increasingly Western medicine, is applied for wound healing, dermatological disorders, and gastrointestinal illness. We investigated the effects of fermented *C. asiatica* extract (FCAE), prepared with *Lactobacillus*, on airway inflammation in a murine model of chronic obstructive pulmonary disease (COPD) induced by cigarette smoke extract (CSE) and lipopolysaccharide (LPS). CSE/LPS stimulation caused marked immune cell infiltration in airways. FCAE (100 and 200 mg/kg) reduced neutrophils in the bronchoalveolar lavage fluid (BALF) by 26.03% and 70.11%, respectively, and decreased activated T cells and B cells in the lung, mediastinal lymph nodes, and Peyer’s patches, while inhibiting collagen fibrosis. FCAE significantly reduced IL-1α (32.51%), CXCL1 (47.63%), CXCL2 (45.37%), and TNF-α (39.51%) levels in the BALF compared with the control group. It also downregulated the expression of muc5ac (58.39%), CXCL1 (67.32%), CXCL2 (57.60%), and TNF-α (54.61%) and suppressed p-STAT3 activation by 50.22%. Furthermore, FCAE enhanced tracheal phenol red secretion by 229.62%, indicating expectorant activity. UPLC analysis identified nine components, which, together with FCAE, inhibited RANTES, TNF-α, and IL-6 in inflammation-induced BEAS-2B cells. Overall, FCAE attenuates immune activation and airway inflammation, supporting its potential as a candidate therapy or functional food for respiratory diseases.

## 1. Introduction

Chronic obstructive pulmonary disease (COPD) is a leading cause of morbidity and mortality worldwide, accounting for over 3 million deaths annually. Its prevalence is rising, particularly in low- and middle-income countries, due to aging populations, smoking, and air pollution [1,2]. Conventional pharmacotherapies, including inhaled bronchodilators and corticosteroids, primarily target symptoms but show limited efficacy in halting disease progression, reversing tissue remodeling, or addressing chronic airway inflammation [3]. Against this backdrop, traditional herbal medicines are gaining renewed interest for their multi-targeted anti-inflammatory properties. Several medicinal plants have demonstrated potential to improve pulmonary function, reduce airway remodeling, and modulate immune responses [4,5].

*Centella asiatica* (family Umbelliferae), traditionally used to treat skin infections by reducing scar tissue formation and accelerating wound healing [6], is widely employed in Asian traditional medicine. *C. asiatica* is rich in triterpenoid compounds, including asiaticoside, madecassoside, asiatic acid, and madecassic acid [7], which exhibit pharmacological activities such as anti-cancer, anti-inflammatory, antioxidant, antimicrobial, and wound-healing effects [8,9,10,11]. Recently, *C. asiatica* extracts have been investigated in respiratory disease models. Extracts of *C. asiatica* have been reported to improve bleomycin-induced lung fibrosis in rats [6]. Although the evidence remains limited, asiatic acid, a major sapogenin-type pentacyclic triterpenoid from *C. asiatica*, has shown protective effects against pulmonary inflammation. In cigarette smoke-induced models, asiatic acid attenuated lung inflammation by modulating cytokine production and suppressing neutrophil infiltration [12]. Moreover, asiatic acid and asiaticoside exhibit anti-inflammatory activity in LPS-induced acute lung injury models, while madecassoside ameliorates bleomycin-induced pulmonary fibrosis in mice [13,14,15].

Terpenoids represent one of the largest and most diverse classes of plant-derived natural products, and they possess substantial industrial and pharmaceutical importance. The development of microbial fermentation-based extraction techniques has been recognized as a promising strategy to enhance the yield, titer, and productivity of these valuable compounds [16].

Fermented herbal extracts can improve the bioactivity, safety, and bioavailability of phytochemicals through microbial transformation [17]. For example, fermented extracts of *C. asiatica* and folic acid using a microorganism mixture significantly enhanced anti-senescence, anti-melanogenic, anti-inflammatory, and skin barrier-enhancing effects [18]. Similarly, *Lactobacillus*-fermented *Sipjeondaebotang*, a traditional East Asian formula, exhibited strong anti-inflammatory activity by suppressing nitric oxide production and pro-inflammatory mediators without cytotoxicity [19]. In addition, fermentation of *Platycodon grandiflorum* extracts increased the content of the active compound platycodin D, which effectively reduced airway inflammation and cough reflex sensitivity in lipopolysaccharide (LPS)/ovalbumin-induced asthma models [20].

These findings suggest that fermented *C. asiatica* extract (FCAE) may represent a promising therapeutic candidate for inflammatory airway diseases such as asthma, COPD, and allergic bronchitis. Therefore, in this study, we investigated the anti-inflammatory effects of FCAE, fermented with *Lactobacillus*, in a murine model of chronic obstructive pulmonary disease (COPD) induced by cigarette smoke extract (CSE) and lipopolysaccharide (LPS). To establish a robust and clinically relevant model of airway inflammation, LPS was combined with CSE to potentiate the inflammatory response and mimic the pathological features of human COPD.

## 2. Results

### 2.1. UPLC Analysis of FCAE Extract

**Figure 1:**
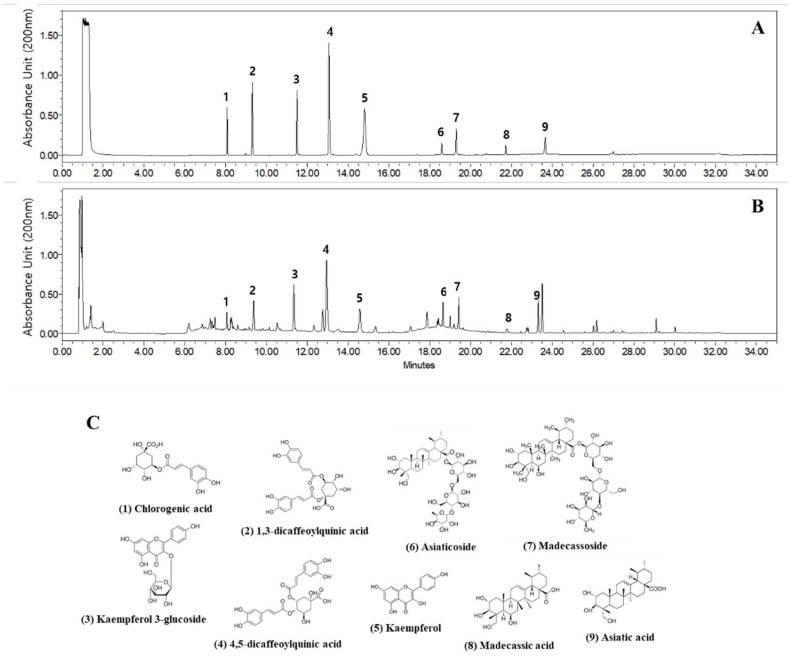
Representative UPLC chromatogram at 200 nm of reference standards (**A**) and FCAE extract (**B**), as well as chemical structures (**C**).

The absorbances of one phenolic compound (chlorogenic acid), two isochlorogenic acids (1,3-dicaffeoylquinic acid and 4,5-dicaffeoylquinic acid), two flavonoids (kaempferol 3-glucoside and kaempferol), and four triterpenoids (asiatic acid, madecassic acid, madecassoside, and asiaticoside) were measured at 200 nm, and the retention times were compared with the reference standards (Figure 1A–C). Each reference standard was dissolved in DMSO at a concentration of 100 mg/mL, and then working solutions at concentrations of 200, 100, and 50 μg/mL were prepared using 70% methanol solution. The FCAE was dissolved in 70% methanol at a concentration of 20 mg/mL, sonicated for 10 min, and filtered through a 0.45 μm PTFE syringe filter before use for UPLC analysis.

### 2.2. Effects of FCAE on Expectoration

**Figure 2:**
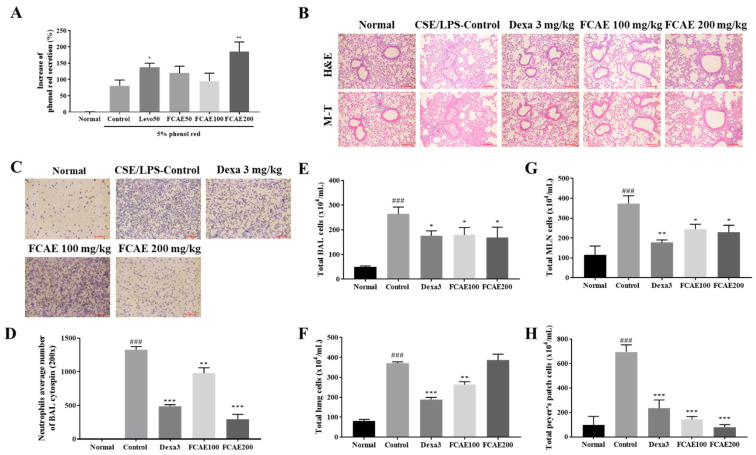
Effects of fermented *C. asiatica* extract (FCAE) on airway inflammation in CSE/LPS-induced COPD mice. (**A**) Expectoration activity by phenol red secretion in the trachea of mice. * *p* < 0.05 and ** *p* < 0.01 (compared to 5% phenol red control). (**B**) Lung histology, (**C**,**D**) representative stained cell image and neutrophil average number by BAL cytospin, and (**E**–**H**) total cell number in CSE/LPS-induced COPD mice. ^###^ *p* < 0.001 (compared to normal), and * *p* < 0.05, ** *p* < 0.01, *** *p* < 0.001 (compared to CSE/LPS-control).

Sputum expectoration is essential to prevent the progression of chronic respiratory diseases [21]. The phenol red secretion assay in murine models is a well-established, rapid, and cost-effective method for quantifying airway mucus secretion [22]. To evaluate the expectorant activity of FCAE, phenol red secretion in the mouse trachea was quantified. Levodropropizine 50 mg/kg (positive control) and FCAE 200 mg/kg significantly increased the secretion of phenol red by 1.70-fold and 2.30-fold, respectively, compared with the 5% phenol red control group (Figure 2A). FCAE at 50 and 100 mg/kg did not show any significant effect.

### 2.3. Effects of FCAE on Inflammatory Response in Airway Inflammation-Induced COPD Mice

Lung histology, inflammation, inflammatory cell infiltration, and collagen fibrosis were increased by CSE/LPS exposure and improved by dexamethasone or FCAE administration (Figure 2B). From the results of cytospin staining of BALF, CSE/LPS exposure increased the number of neutrophils in the BALF, and the number of cells decreased in the dexamethasone- or FCAE-treated mice (Figure 2C,D).

### 2.4. Effects of FACE on Airway Immune Cell Number in BALF, Lung, MLN, and Peyer’s Patch

To investigate airway inflammation, airway immune cells in BALF, lungs, MLN, and Peyer’s patches were counted using fluorescence-activated cell sorting (FACS). First, the total number of immune cells in tissues was counted. CSE/LPS exposure of the mice increased the total cells from the lungs, MLN, Peyer’s patches, and BALF, and these increased cells were decreased by oral administration of dexamethasone and FCAE (Figure 2E–H). FACS analysis showed that the number of immune cells, such as neutrophils, increased in the BALF and lungs following CSE/LPS exposure, and decreased following dexamethasone or FCAE administration (Table 1). In particular, (CD4^+^, CD8^+^, CD4^+^/CD69^+^, and CD62L^−^/CD44high^+^) T cells; CD21/CD35^+^B220^+^-activated B cells; and Gr-1^+^/SiglecF^−^ neutrophils dramatically increased in CSE/LPS-exposed mice and decreased in dexamethasone- or FACE-administered mice (Table 1). The number of CD3^+^ T cells, CD19^+^ B cells, and CD4^+^CD44^+^ T cells in the MLN was increased by CSE/LPS exposure and decreased by dexamethasone or FCAE administration. The numbers of CD4^+^ T cells, CD8^+^ T cells, and CD4^+^/B200^+^ B cells in Peyer’s patches were increased by CSE/LPS exposure and decreased by dexamethasone or FCAE administration. These results indicated that FCAE ameliorated the airway inflammatory response in CSE/LPS-exposed COPD mice.

### 2.5. Effects of FCAE on Expression of Inflammatory Mediators in BALF and Lung

**Figure 3:**
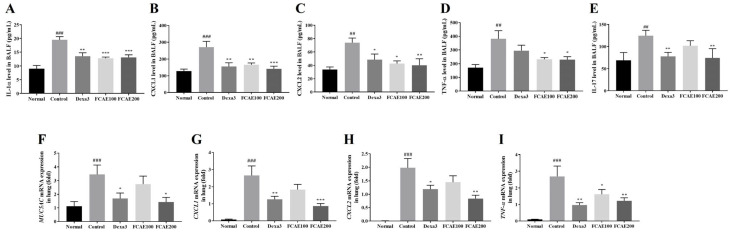
Effect of fermented *C. asiatica* extract (FCAE) on cytokine/chemokine expression in BALF and lung in CSE/LPS-induced COPD mice. (**A**–**E**) Cytokine/chemokine levels in BALF by ELISA and (**F**–**I**) cytokine/chemokine mRNA gene expression in lung by real-time RT-PCR. ^##^ *p* < 0.01, ^###^ *p* < 0.001 (compared to normal), and * *p* < 0.05, ** *p* < 0.01, *** *p* < 0.001 (compared to CSE/LPS-control).

**Figure 4:**
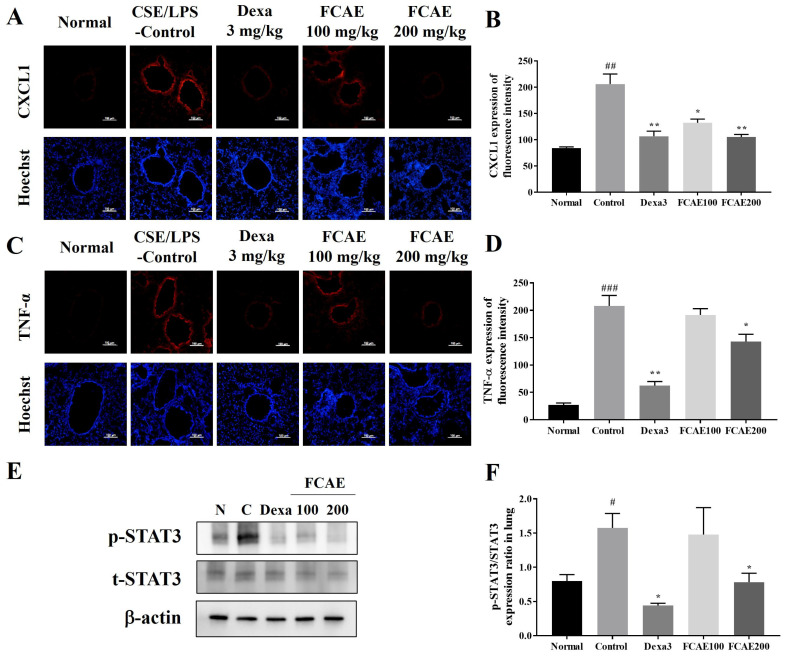
Effect of fermented *C. asiatica* extract (FCAE) in lung in CSE/LPS-induced COPD mice. (**A**,**B**) CXCL1 immunohistofluorescence, (**C**,**D**) TNF-α immunohistofluorescence, and (**E**,**F**) STAT3 expression by Western blot analysis. ^#^ *p* < 0.05, ^##^ *p* < 0.01, ^###^ *p* < 0.001 (compared to normal), and * *p* < 0.05, ** *p* < 0.01 (compared to CSE/LPS-control).

CXCL2, IL-1α, CXCL1, IL-17, and TNF-α levels in BALF were increased in CSE/LPS-exposed mice and decreased in the dexamethasone or FCAE-treated mice (Figure 3A–E). In the lung, expression of MUC5AC, TNF-α, CXCL1, and CXCL2 mRNA increased by CSE/LPS airway exposure and decreased by dexamethasone or FCAE 200 mg/kg administration (Figure 3F–I). From the results of lung fluorescence staining, CXCL1 and TNF-α expression increased in the CSE/LPS mice and decreased in the dexamethasone or FCAE mice (Figure 4A–D). Western blot analysis of the lung showed that p-STAT3 protein, a major transcription factor in the inflammatory response, was increased by CSE/LPS exposure and decreased by dexamethasone or FCAE administration (Figure 4E,F and Figure S1).

### 2.6. Effects of FCAE and Constituents on Inflammatory Response in BEAS-2B Cells

**Figure 5:**
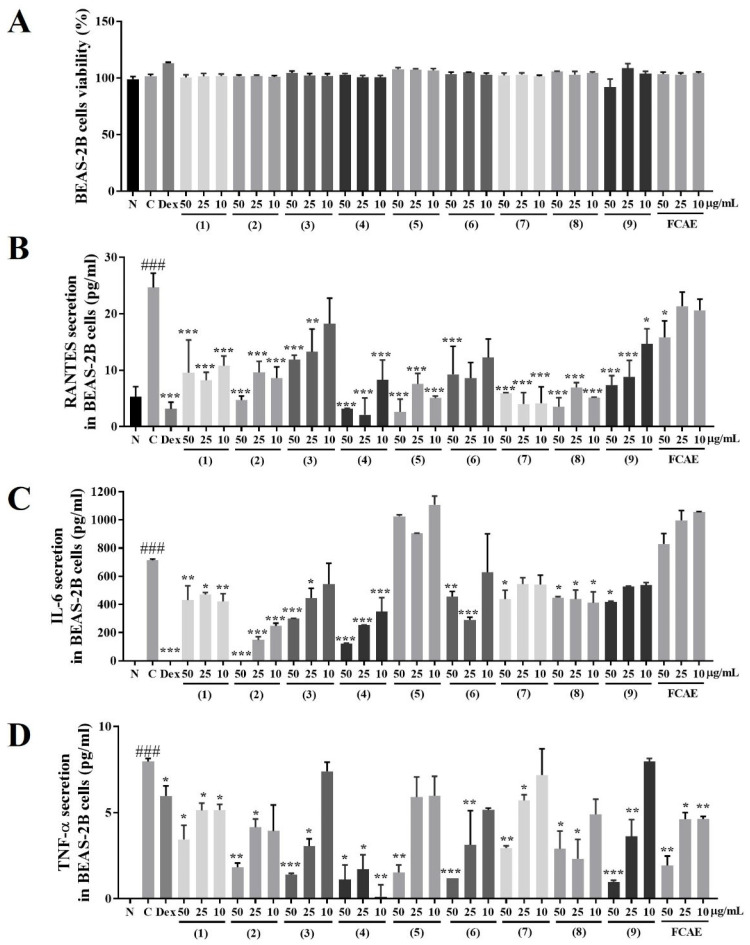
Effects of fermented *C. asiatica* extract (FCAE) and constituents in LPS-induced human bronchial epithelial cells (BEAS-2B). (**A**) Cell viability and LPS-induced; (**B**) RANTES; (**C**) IL-6; or (**D**) TNF-α secretion. ^###^ *p* < 0.01 (compared to normal), and * *p* < 0.05, ** *p* < 0.01, *** *p* < 0.001 (compared to LPS-control). (1) Chlorogenic acid, (2) 1,3-dicaffeoylquinic acid, (3) kaempferol 3-glucoside, (4) 4,5-dicaffeolyquinic acid, (5) kaempferol, (6) asiaticoside, (7) madecassoside, (8) madecassic acid, and (9) asiatic acid.

The effects of FCAE and its constituents on airway inflammation were investigated in LPS-induced BEAS-2B cells. FCAE and its constituents (10–50 µg/mL concentration) did not exhibit cell toxicity in BEAS-2B cells (Figure 5A). RANTES levels in the cell supernatants were increased by TNF-α and decreased by FCAE and treatment with nine compounds (Figure 5B). IL-6 and TNF-α levels in the cell supernatants were increased by LPS and decreased by FCAE and nine compounds (Figure 5C,D). These results demonstrated that the nine compounds may contribute to the inhibitory effect of FCAE on airway inflammation by suppressing inflammatory cytokines.

## 3. Discussion

The effects of *C. asiatica* on respiratory diseases have been reported to include improvements in COPD, pulmonary fibrosis, acute lung injury, and lung cancer in both mouse models and cell systems, with asiatic acid and asiaticoside identified as the major active anti-inflammatory components [23,24]. Fermentation of medicinal herbs is an important processing strategy to enhance therapeutic efficacy and/or generate novel effects while reducing toxicity [25,26]. In our preliminary work, fermentation of *C. asiatica* extract with *Lactobacillus* enhanced in vitro anti-inflammatory activity (Supplementary Materials, Figure S2). Fermented extracts of *C. asiatica* (FCAE) exhibited superior biological activity compared with non-fermented extracts (CAE), as evidenced by a greater suppression of RANTES and IL-6 production in TNF-α- or LPS-stimulated BEAS-2B cells. Therefore, in this study, we evaluated the anti-inflammatory effects of FCAE in a CSE/LPS-induced COPD mouse model, along with a comprehensive chemical profiling of FCAE.

Airway responses to inflammation are associated with increased mucus secretion [27]. Effective expectoration constitutes a fundamental host defense mechanism in the respiratory tract, facilitating the clearance of mucus, microbial pathogens, and inhaled particulates; this process is essential for maintaining pulmonary hygiene and slowing the progression of chronic respiratory diseases such as COPD and bronchiectasis [21]. In the phenol red mouse model used to investigate expectorant activity, FCAE significantly increased tracheal phenol red secretion, supporting its expectorant effect. MUC5AC, the major glycoprotein component of mucus encoded by the conserved muc5ac gene, is expressed in airway epithelial cells and mediates IL-17A-induced mucus cell hyperplasia and mucus production via STAT3 signaling, thereby contributing to airway obstruction and inflammation [27,28,29,30].

In chronic COPD inflammation, the predominant airway immune cells are macrophages, B cells, T cells, and particularly neutrophils. These activated cells release proteases, cytokines, and chemokines such as matrix metalloproteinases, TNF-α, IL-6, IL-17, CXCL8/IL-8, CXCL1, CXCL2, RANTES, and CCL2/MCP-1, which collectively augment inflammation, apoptosis, airway injury, and lung function decline via STAT3 signaling [31,32,33]. The present study demonstrated that FCAE inhibited IL-1α, IL-17, MUC5AC, TNF-α, IL-6, CXCL1, CXCL2, and STAT3 phosphorylation, while reducing neutrophils, T cells, and B cells in the BALF and lungs of CSE/LPS-induced COPD mice. These findings indicate that FCAE regulates mucus secretion and airway inflammation through STAT3 downregulation.

Previous studies have consistently shown that suppression of STAT3 signaling plays a pivotal role in alleviating airway inflammation. Inhibition of STAT3 has been reported to reduce eosinophilic infiltration, cytokine release, and airway hyperresponsiveness in asthma models, while also attenuating IL-6-mediated inflammatory signaling and airway remodeling in COPD [34,35,36]. Moreover, pharmacological or mitochondrial inhibition of STAT3 effectively limits the activation of immune cells, such as Th2 and ILC2, thereby reducing chronic inflammatory responses in the lung [37]. Together, these findings support that downregulation of STAT3 contributes to the resolution of airway inflammation. In this context, the observed effects of FCAE—enhanced triterpenoid accumulation and subsequent STAT3 inhibition—suggest that fermented *C. asiatica* extracts may act as a modulator of airway inflammation by suppressing excessive mucus secretion and inflammatory signaling.

To examine whether FCAE influences intestinal immune modulation, immune cells in MLN and Peyer’s patches—key inductive sites of intestinal immunity—were assessed by FACS. FCAE treatment reduced both T and B cell populations, suggesting that FCAE attenuates airway inflammation partly through intestinal immune regulation.

UPLC analysis identified nine compounds in FCAE, including phenylpropanoids [chlorogenic acid (5-O-caffeoylquinic acid), 1,3-di-O-caffeoylquinic acid, 4,5-di-O-caffeoylquinic acid], flavonols and glycosides [kaempferol, kaempferol-3-O-β-D-glucopyranoside (astragalin)], pentacyclic triterpenoid saponins [madecassoside, asiaticoside], and pentacyclic triterpenoid aglycones [asiatic acid, madecassic acid].

Previous studies have reported that *Artemisia gmelinii* extract, including chlorogenic acid and caffeoylquinic acid as the major components, attenuated lung inflammation in CSE/LPS-treated mice via inhibition of the MAPK and NF-κB pathways [38]. Astragalin was also shown to alleviate LPS- and cigarette smoke-induced lung inflammation in mice [39,40]. Moreover, asiatic acid and asiaticoside exert anti-inflammatory effects in LPS-induced acute lung injury models by suppressing cytokine production through inhibition of the NF-kB signaling pathway [13,14], while madecassoside ameliorated bleomycin-induced pulmonary fibrosis in mice by inhibiting TGF-β1 signaling [15]. In addition, madecassic acid has been reported to potently suppress inflammatory mediators, including nitric oxide, TNF-α, and IL-6 in LPS-stimulated RAW 264.7 macrophage cells [41].

In the present study, these compounds collectively reduced the secretion of RANTES, TNF-α, and IL-6 in inflammation-induced BEAS-2B airway epithelial cells, suggesting that they might contribute to the anti-inflammatory activity of FCAE in CSE/LPS-induced COPD. Taken together, these results indicate that FCAE exerts its protective effects on the airway through a multifactorial mechanism involving the enhancement of triterpenoid biosynthesis, inhibition of STAT3 and NF-κB signaling, and regulation of mucus secretion. Given the central roles of these pathways in chronic airway diseases, FCAE and its bioactive constituents hold promise as potential therapeutic candidates for the prevention or treatment of COPD and related airway disorders.

Nevertheless, this study has several limitations. The precise molecular targets of individual compounds were not fully elucidated. In addition, synergistic interactions among the multiple bioactive compounds of FCAE remain to be clarified in in vivo COPD models. Future studies employing animal experiments and pathway-specific approaches are warranted to validate these findings and to define the comprehensive mechanisms underlying FCAE-mediated protection against airway inflammation.

## 4. Materials and Methods

### 4.1. Materials

Ethanol, acetonitrile, methanol, and water were of HPLC grade (J.T. Baker, Phillipsburg, NJ, USA). Phosphoric acid, kaempferol, and kaempferol glucoside were purchased from Sigma-Aldrich (St. Louis, MO, USA). The reference standards, chlorogenic acid, 1,3-dicaffeoylquinic acid, 4,5-dicaffeoylquinic acid, madecassoside, asiaticoside, madecassic acid, and asiatic acid were purchased from Chemfaces (Wuhan, China).

### 4.2. UPLC Analysis Fermentation and Extraction of Centella asiatica

Dried *Centella asiatica* leaves were obtained from Shaanxi EDW Biotech Co., Ltd. (Xi’an, China). The leaves (500 g) were sterilized at 120 °C for 30 min after adding 2.5 L of distilled water. After equilibrating to room temperature, 1L of *Lactobacillus plantarum* (1 × 104 cfu/mL) was added and cultured at 35 °C for 3 h, evaporated while heating at 50 °C in a vacuum (EYELA, Tokyo, Japan), and freeze-dried to produce fermented *C. asiatica* extract (FCAE). The FCAE was produced 3 times under the same conditions, with an extraction yield of 29.7~36.4%. Asiatic acid, the marker compound of FCAE, was detected at 1.00 ± 0.21 mg/g.

### 4.3. UPLC Analysis

An Ultra-Performance Liquid Chromatography (UPLC, Waters, MA, USA) System equipped with a quaternary pump, auto-sampler, and photodiode array detector with Acquity UPLC^®^ BEH C18 (Waters, Milford, FL, USA), 100  ×  2.1 mm, 1.7 μm, was used for analysis. A binary gradient was applied using solvent A (0.1% phosphoric acid in water) and solvent B (acetonitrile) at a flow rate of 0.3 mL/min with the following elution program: 0–2 min, 5–5% B; 12–13 min, 25–42% B; 20–22 min, 52–65% B; 28–30 min, 82–100% B; and 32–34 min, 10–10% B. Detection was performed at 200 nm. The column was maintained at 40 °C with an injection volume of 2 µL.

### 4.4. Expectoration Activity

Expectoration activity was determined using the phenol red secretion method as described previously [42]. The study was approved by the Committee for Animal Ethics of the Korea Institute of Oriental Medicine (code 23-054). A total of 72 mice were separated into 6 groups (*n* = 12/group): normal, control, positive control, and 3 experiment groups. A dose of 50 mg/kg of levodropropizine (Levosol; PharmGen Science, Seoul, Republic of Korea) was used as a positive control. The fermented *C. asiatica* extract (FCAE) was orally administered at 50, 100, and 200 mg/kg. Vehicle was administered to both normal and control mice. After drug administration for 3 days, mice in all groups, except for the normal group, were injected intraperitoneally with 5% phenol red. After 60 min, the trachea was removed and soaked in 1 mL saline for 1 h to extract phenol red. The reaction was stopped by addition of 0.1 mL sodium hydroxide, and the amount of phenol red secreted at 546 nm was measured using a microplate reader.

### 4.5. Airway Inflammatory Animal Experiments

All animal experiments were approved by the Institutional Animal Care and Use Committee of Daejeon University (ethical approval code DJUARB2022-032) and conducted in accordance with the committee guidelines. Under controlled specific pathogen-free conditions (21 °C ± 2 °C temperature, 60% ± 10% humidity), male BALB/c mice (7 weeks old, Orient Bio, Seongnam, Republic of Korea) were acclimated. Airway inflammation was caused by intranasal administration of a mixture of 4 mg/mL cigarette smoke extract (CSE) and 100 μg/mL lipopolysaccharide (LPS; Sigma-Aldrich, St. Louis, MO, USA) to mice according to a previously described method [43]. CSE was kindly provided by Dr. Lim (Chungbuk National University, Cheongju, Republic of Korea) [44]. The 60 mice were assigned to 5 groups (*n* = 12/group) as follows: (1) normal, (2) CSE plus LPS-control, (3) CSE plus LPS-Dexa 3 mg/kg, (4) CSE plus LPS-FCAE 100 mg/kg, or (5) CSE plus LPS-FCAE 200 mg/kg. The normal group received saline, and all other groups were intranasally exposed to CSE/LPS once every 7 days. Dexamethasone and FCAE were dissolved in a vehicle (saline) and administered orally daily for 14 days. The same volume of vehicle was administered to the normal and control mice.

### 4.6. Bronchoalveolar Lavage Fluid (BALF) and Lung Cell Collection

BALF was collected via tracheal cannulation, and cells were obtained using Cytospin and stained with Diff-Quick solution for morphological evaluation. The cells from lung were isolated by reacting with 2 mg/mL dispase and 1 mg/mL collagenase IV (Sigma-Aldrich, St. Louis, MO, USA) for 30 min at 37 °C.

### 4.7. Fluorescence-Activated Cell Sorting (FACS) Analysis

Cells collected from the bronchoalveolar lavage fluid (BALF), lungs, and lung mediastinal lymph nodes (MLNs) were subjected to FACS analysis according to a previously described method [43]. Peyer’s patch cells were incubated with immunoglobulin G (IgG) antibodies against CD4 (RM4-5, rat IgG2a), CD8 (53-6.7, rat IgG2a), and B200 (RA3-6B2, rat IgG2a), and the cell analysis was performed with a FACSCaliber (BD Biosciences, San Diego, CA, USA).

### 4.8. Histopathological Examination

Lung tissue was harvested and fixed for 24 h, followed by paraffin embedding. Sections (6 μm) were prepared and stained with hematoxylin and eosin (H&E) and Masson’s trichrome (M-T) (Sigma-Aldrich) to investigate the inflammation, inflammatory cell infiltration, and fiber formation and observed by light microscopy. The degree of inflammation was scored on a subjective scale ranging from 0 to 2, according to previously reported criteria [45].

### 4.9. Enzyme-Linked Immunosorbent Assay (ELISA)

The levels of IL-17, IL-1α, TNF-α, CXC motif chemokine ligand (CXCL) 1, and CXCL2 (macrophage inflammatory protein-2; MIP-2) were measured from the BALF using ELISA kits (R&D, Minneapolis, MN, USA). Also, IL-6, TNF-α, and RANTES (R&D, Minneapolis, MN, USA) levels from the cell culture supernatant were measured.

### 4.10. Quantitative Real-Time RT-PCR Analysis

Total RNA was extracted from lung tissue using RNAsolB reagent (Tel-Test, Friendswood, TX, USA). The expression levels of TNF-α, CXCL2, CXCL1, and MUC5AC were quantified using a 7500 Fast Real-Time PCR system (Applied Biosystems, Foster City, CA, USA) with SYBR Green PCR Master mix and gene-specific primers. Primers were prepared as previously reported [43].

### 4.11. Immunohistofluorescence Staining

Lung was embedded in optimal cutting temperature compound and sectioned to 20 μm thickness using a cryostat microtome (Leica Microsystems, Wetzlar, Germany). The sections were fixed in 4% formaldehyde for 45 min, permeabilized in nonyl phenoxypolyethoxylethanol-40 (0.5%), and blocked with 2.5% bovine serum albumin/horse serum for 16 h. The sections were incubated overnight at 4 °C with antibodies against CXCL1 or TNF-α and subsequently incubated with a fluorescein-labeled secondary antibody for 2 h. After Hoechst staining of the nucleus, the fluorescence images were obtained using a fluorescence microscope (Nikon Instruments Inc., Mississauga, ON, Canada)

### 4.12. Western Blot

Total protein was extracted from lung tissue using the PRO-PREP kit (iNtRON, Seoul, Republic of Korea). Proteins were electrophoresed using 4–15% gradient SDS-PAGE gel and transferred onto a membrane. The membrane was blocked for 60 min by EzBlock Chemi (ATTO, Koto, Japan), followed by incubation with anti-STAT3, phospho-anti-STAT3, and β-actin antibodies (Cell signaling, Danvers, MA, USA) for 24 h at 4 °C. β-actin acted as the internal control. The membrane was then incubated with a secondary antibody (Cell Signaling, Danvers, MA, USA) for 1 h. The bands were imaged using chemiluminescence (Supex, Daegu, Republic of Korea) and analyzed using ImageJ 1.52a software (Washington, DC, USA).

### 4.13. Cell Culture

Human bronchial epithelial BEAS-2B cells (ATCC, Manassas, VA, USA) were cultured in Dulbecco’s modified Eagle’s medium (Gibco, New York, NY, USA) containing 10% fetal bovine serum and 1% penicillin–streptomycin at 37 °C in a humidified incubator with 5% CO_2_. Inflammation was induced by stimulating the cells with 20 µg/mL LPS (Sigma-Aldrich).

The cells (5 × 10^4^ cells/well) were seeded in 96-well plates and treated with the compounds at 10, 25, or 50 μg/mL with or without 20 µg/mL LPS for 24 h. DMSO (at a concentration of 0.1%) was used as a control and dissolved the samples. To measure cell viability, Cell Counting Kit-8 reagent (Dojindo, Kumamoto, Japan) was treated for 2 h and absorbance was evaluated at 450 nm. The cell supernatants were used for ELISA (R&D) to measure the cytokines RANTES, IL-6, and TNF-α.

### 4.14. Statistical Analysis

Data were analyzed using one-way analysis of variance (ANOVA), followed by Dunnett’s multiple comparison test, using SPSS version 14.0. The results are expressed as the mean ± SEM for animal studies or mean ± SD for cell studies, with significant differences denoted as follows: # *p* < 0.05, ## *p* < 0.01, and ### *p* < 0.001 (compared to normal) and * *p* < 0.05, ** *p* < 0.01, and *** *p* < 0.001 (compared to CSE/LPS- or LPS-control).

## 5. Conclusions

In conclusion, fermented *Centella asiatica* extract (FCAE), prepared with *Lactobacillus*, alleviated airway inflammation and lung injury in CSE/LPS-induced COPD mice. FCAE reduced the infiltration of inflammatory cells in BALF, lung, and associated lymphoid organs, while also suppressing cytokine and chemokine expression and inhibiting STAT3 activation. In addition, FCAE exhibited expectorant activity by enhancing tracheal phenol red secretion. UPLC analysis revealed nine major components, including phenolic acids and triterpenoids, which collectively inhibited RANTES, TNF-α, and IL-6 in inflammation-induced BEAS-2B cells. These findings suggest that FCAE has potential as a therapeutic or functional food candidate for the prevention and management of chronic airway inflammatory diseases such as COPD.

## Figures and Tables

**Table 1:** The effects of fermented *C. asiatica* extract (FCAE) on airway immune cell number and neutrophilic airway inflammation in the CSE/LPS-induced COPD murine model.

Cell Phenotypes in Lung, BALF, MLN, and Peyer’s Patch		CSE/LPS-Induced COPD Murine Model (Absolute No.)				
		Normal	CSE/LPS-Control	CSE/LPS-Dexa 3 mg/kg	CSE/LPS-FCAE 200 mg/kg	CSE/LPS-FCAE 100 mg/kg
Lymphocyte (×10^6^ cells)	Lung	37.51 ± 6.29	27.90 ± 6.84	22.54 ± 2.14	59.72 ± 5.36 **	37.06 ± 3.95
Neutrophils (×10^6^ cells)		24.84 ± 2.30	248.57 ± 4.51 ^###^	107.32 ± 5.74 ***	191.27 ± 18.76 **	143.78 ± 7.00 ***
Eosinophils (×10^5^ cells)		14.18 ± 1.10	87.57 ± 4.33 ^###^	54.73 ± 6.50 ***	116.39 ± 9.80 **	74.00 ± 4.22 *
CD4^+^ (×10^6^ cells)		31.98 ± 4.16	116.97 ± 2.70 ^###^	68.77 ± 5.83 ***	135.90 ± 8.75 *	91.26 ± 7.15 **
CD8^+^ (×10^6^ cells)		15.73 ± 2.20	53.81 ± 5.47 ^###^	32.61 ± 1.61 **	68.71 ± 7.51	43.97 ± 2.87
CD4^+^/CD69^+^ (×10^5^ cells)		1.75 ± 0.27	32.42 ± 7.40 ^###^	12.89 ± 3.20 *	20.67 ± 2.51	20.49 ± 5.00
CD62L^−^/CD44^high+^ (×10^6^ cells)		4.98 ± 0.61	151.05 ± 8.97 ^###^	41.93 ± 2.45 ***	101.72 ± 11.17 **	80.90 ± 4.79 ***
CD21/CD35^+^B220^+^ (×10^5^ cells)		0.67 ± 0.08	6.39 ± 0.48 ^###^	1.82 ± 0.49 ***	3.26 ± 0.94 **	1.84 ± 0.30 ***
Gr-1^+^SiglecF^−^ (×10^6^ cells)		8.99 ± 0.47	251.88 ± 6.63 ^###^	86.38 ± 1.38 ***	129.31 ± 14.26 ***	110.22 ± 12.93 ***
Lymphocyte (×10^6^ cells)	BALF	1.36 ± 0.28	10.35 ± 0.97 ^###^	6.04 ± 1.13 **	6.71 ± 0.87 **	7.39 ± 0.97 *
Neutrophils (×10^6^ cells)		27.77 ± 5.62	237.61 ± 24.44 ^###^	154.12 ± 18.81 **	147.93 ± 40.88	154.92 ± 27.48 *
Eosinophils (×10^5^ cells)		16.12 ± 3.26	14.58 ± 3.26	14.83 ± 1.32	12.22 ± 2.32	15.49 ± 2.14
CD4^+^ (×10^6^ cells)		1.79 ± 1.79	85.13 ± 9.59 ^###^	48.67 ± 5.58 **	40.31 ± 6.20 **	47.26 ± 7.13 **
CD8^+^ (×10^6^ cells)		2.56 ± 1.56	65.78 ± 20.34 ^##^	32.59 ± 5.44	39.83 ± 9.65	42.22 ± 8.54
CD4^+^/CD69^+^ (×10^5^ cells)		6.69 ± 6.23	34.73 ± 4.53 ^##^	16.40 ± 1.63 **	10.38 ± 3.01 ***	9.22 ± 2.99 ***
CD62L^−^/CD44^high+^ (×10^6^ cells)		2.78 ± 0.76	173.96 ± 23.65 ^###^	108.42 ± 17.36 *	96.82 ± 24.47 *	101.21 ± 27.80 *
Gr-1^+^SiglecF^−^ (×10^6^ cells)		0.24 ± 0.03	234.65 ± 23.45 ^###^	139.50 ± 15.30 **	127.66 ± 27.48 **	155.12 ± 26.28 *
CD3^+^ (×10^6^ cells)	MLN	57.88 ± 23.01	250.14 ± 35.88 ^###^	90.20 ± 6.78 ***	133.74 ± 19.14 **	149.97 ± 11.43 **
CD19^+^ (×10^6^ cells)		48.06 ± 17.66	106.07 ± 2.65 ^##^	78.70 ± 8.95 **	85.79 ± 14.97	78.39 ± 12.38 *
CD4^+^CD44^+^ (×10^6^ cells)		6.47 ± 2.10	36.27 ± 4.40 ^###^	14.49 ± 0.78 ***	17.06 ± 3.77 **	19.84 ± 1.59 **
CD4^+^ (×10^6^ cells)	Peyer’s patch	31.25 ± 23.13	152.39 ± 16.42 ^###^	47.22 ± 12.86 ***	19.14 ± 5.28 ***	31.24 ± 7.32 ***
CD8^+^ (×10^6^ cells)		24.84 ± 2.30	248.57 ± 4.51 ^##^	10.732 ± 5.74	183.64 ± 22.45 ***	198.41 ± 16.28 ***
CD4^+^/B200^+^ (×10^6^ cells)		14.18 ± 1.10	87.57 ± 4.33 ^###^	54.73 ± 6.50 ***	105.69 ± 9.28 ***	129.50 ± 25.35 ***

^##^ *p* < 0.01, ^###^ *p* < 0.001 (compared to normal), and * *p* < 0.05, ** *p* < 0.01, *** *p* < 0.001 (compared to CSE/LPS-control).

## Data Availability

Data is contained within the article or Supplementary Material.

## Supplementary Materials

The following supporting information can be downloaded at https://www.mdpi.com/article/10.3390/plants14223416/s1, Figure S1: Original Western blot images. Figure S2: Effect of CAE and FCAE in BEAS-2B cells.

## Abbreviations

The abbreviations used in this manuscript are as follows:

FCAE	Fermented *Centella asiatica* extract
COPD	Chronic Obstructive Pulmonary Disease
CSE	Cigarette Smoke Extract
LPS	BALF Lipopolysaccharide
BALF	Lipopolysaccharide
UPLC	Ultra-Performance Liquid Chromatography
TNF-α	Tumor Necrosis Factor alpha
IL-6	Interleukin-6
STAT3	Signal Transducer and Activator of Transcription 3
RANTES	Regulated upon Activation, Normal T Cell Expressed and Presumably Secreted
BEAS-2B	Human bronchial epithelial cell line
