# Supplementary material for: Lactobacillus-Fermented Centella asiatica Extract Inhibits Airway Inflammation in Cigarette Smoke Extract/LPS-Induced Mice
Source: Plants (Basel). 2025 Nov 7;14(22):3416. doi: 10.3390/plants14223416 (PMC12655661; doi:10.3390/plants14223416)
Supplement: Supplementary file 1 [file plants-14-03416-s001.zip › plants-3892466-supplementary.pdf]

Supplementary Materials

Supplementary method 1. *Effect of CAE and FCAE on inflammatory cytokine/chemokine secretion in BEAS-2B cells*

Human bronchial epithelial cell line (BEAS-2B) (ATCC, Manassas, VA, USA) was grown in Dulbecco's modified Eagle's medium (Gibco, New York, NY, USA) with 10% fetal bovine serum (FBS) and 1% penicillin–streptomycin at 37 °C in a 5% CO_2 and 95% humidified atmosphere. BEAS-2B cells were stimulated with 10 ng/mL TNF-α (R&D systems) or 100 ng/mL LPS (Sigma-Aldrich Co., St. Louis, MO, USA) in the absence or presence of CAE or FCAE (10, 25, 50, or 100 μg/mL) for 24 h. Dexamethasone (Sigma-Aldrich) 1 μM was used as positive control. Human RANTES and IL-6 levels were measured from the cell culture supernatant using enzyme-linked immunosorbent assays (ELISA) kits (R&D Systems, Minneapolis, MN, USA) and the absorbance was also observed at 450 nm (SpectraMax; Molecular devices, San Jose, CA, USA).

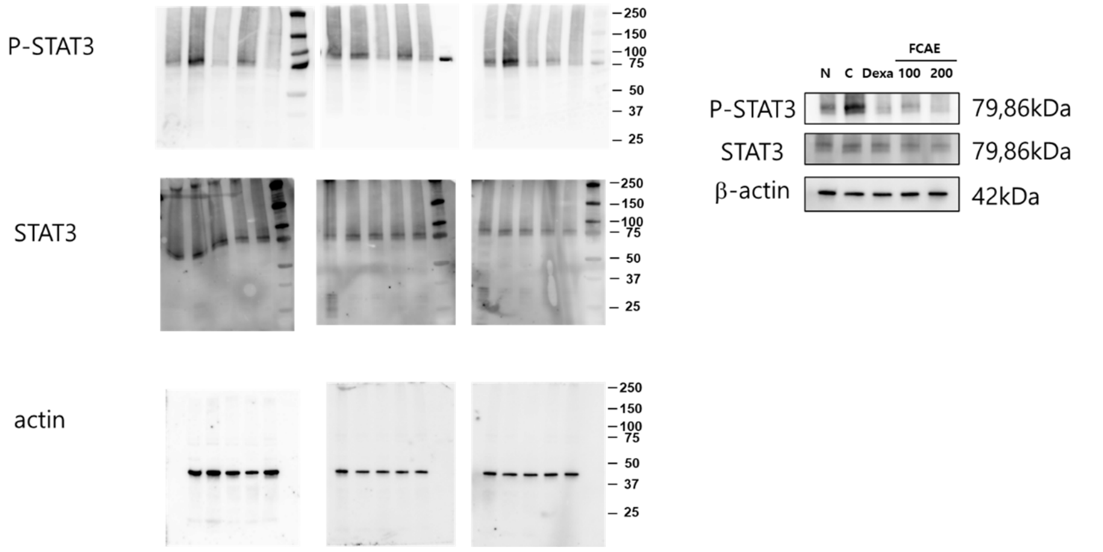

Figure S1. Original western blot images in lung.

(A)

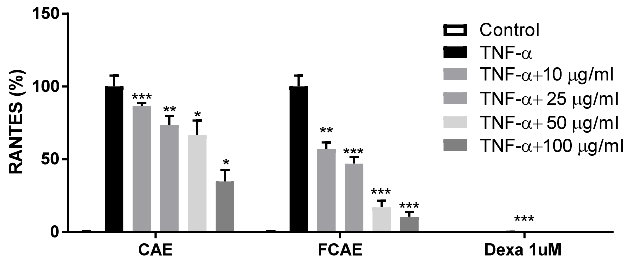

(B)

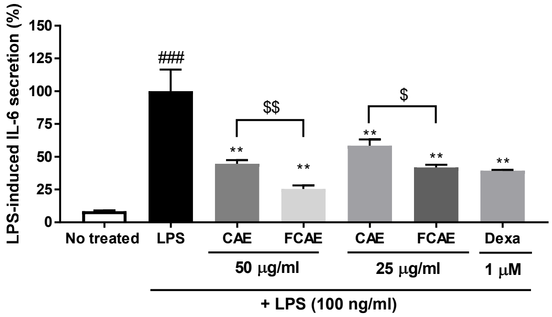

Figure S2. Effect of CAE and FCAE in BEAS-2B cells (comparison). BEAS-2B cells were stimulated with 10 ng/mL TNF-α or 100 ng/mL LPS in the absence or presence of CAE or FCAE (10, 25, 50, or 100 μg/mL) for 24 h. Human RANTES and IL-6 levels were measured using ELISA. ### $p < 0.001$ compared with the no treated group. * $p < 0.05$, ** $p < 0.01$, *** $p < 0.001$ compared with the TNF-α or LPS-treated group. $ $p < 0.05$, $$ $p < 0.01$ compared between CAE and FCAE. Dexa, dexamethasone.
